# Effects of cataract surgery and intra-ocular lens implantation on visual function and quality of life in age-related cataract patients: a systematic review protocol

**DOI:** 10.1186/s13643-019-1113-6

**Published:** 2019-08-13

**Authors:** Shalu Jain, Kavitha Rajshekar, Anjana Aggarwal, Akshay Chauhan, Vijay Kumar Gauba

**Affiliations:** 1 Health Technology Assessment in India, Department of Health Research, Ministry of Health and Family Welfare, Indian Red Cross Society Building, 1, Red Cross Road, New Delhi, 110001 India; 2 Indian Council of Medical Research, (ICMR), New Delhi, India

**Keywords:** Cataract, Health-related quality of life, Visual function, Health technology assessment, Economic evaluations, Cost effectiveness

## Abstract

**Background:**

Cataract is the leading cause of blindness and low vision worldwide. Presently, cataract surgery is the only treatment for cataract and is very effective in restoring sight. In cataract surgery, the natural lens of the eye that becomes clouded is removed and replaced with an artificial intraocular lens. There are multiple techniques for removal of lens as well as many types of intraocular lenses available for implantation. For this reason, it becomes imperative to monitor the impact of different surgical techniques and different intraocular lenses on health-related quality of life (HRQoL) of the patients. This systematic review aims to evaluate HRQoL evidences on effects of different types of cataract surgeries and intraocular lenses on visual function and quality of life in age-related cataract patients.

**Method:**

Databases like Cochrane, EMBASE, SCOPUS, NHS Economic Evaluation Database (NHS EED), Health Technology Assessment (HTA) database, MEDLINE, ClinicalTrials.gov, Current Controlled Trials and World Health Organization International Clinical Trials Registry Platform (WHO ICTRP) will be searched systematically. Two reviewers will independently screen studies using predefined inclusion and exclusion criteria along with the extraction of data, and assessment of methodological quality using a standard checklist.

**Discussion:**

This systematic review will help in understanding how different types of cataract surgeries and intraocular lenses make a difference on quality of life of age-related cataract patients in terms of visual function and health-related quality of life. As the review attempts to bring together all the cataract-related HRQoL evidences pertaining to different cataract surgical techniques, different intraocular lenses and cataract-related complications, it will also identify gaps in evidence.

**Systematic review registration:**

PROSPERO CRD42018092377

**Electronic supplementary material:**

The online version of this article (10.1186/s13643-019-1113-6) contains supplementary material, which is available to authorized users.

## Background

Health Technology Assessment (HTA) is an internationally accepted tool to inform decision making for better management of existing resources for Universal Health Coverage (UHC) [1–3]. Health Technology Assessment in India (HTAIn) has been institutionalised in January 2017 under the Department of Health Research (DHR), Ministry of Health and Family Welfare (MoHFW) by the Government of India to facilitate the process of transparent and evidence-based decision-making for improved healthcare delivery [4]. HTAIn is entrusted with the responsibility to analyse evidences related to cost-effectiveness, clinical-effectiveness and equity issues regarding the deployment of health technologies like new medicines, devices and health programmes by means of HTA studies, which will in turn help in the efficient use of a limited health budget and provide people access to quality healthcare at minimum costs [5, 6].

The first HTA topic selected for study at HTAIn secretariat was ‘Health Technology Assessment of intraocular lenses for treatment of age-related cataracts’. The request for this topic came from Rashtriya Swasthya Bima Yojana (RSBY), a Government health insurance scheme that has now been subsumed by the National Health Protection Scheme (NHPS) under Ayushman Bharat Mission [7, 8]. Under this HTA study, five individual literature reviews were conducted to gather the evidences namely on clinical effectiveness, cost effectiveness, health-related quality of life (HRQoL), costing and equity. The present study (systematic literature review on HRQoL) is a part of the HTA study on ‘Health Technology Assessment of intraocular lenses for treatment of age-related cataracts’.

Cataract is a condition in which the lens of the eye becomes clouded preventing clear vision [9]. Cataract is the leading cause of blindness (51%) and low vision (33%) worldwide [10]. When vision < 20/200 in the better eye on presentation is defined as blindness, it has also been reported that cataract is responsible for 50–80% of the bilaterally blind in the country [11–16].

Presently, cataract surgery is the only treatment for cataract, with high success rates, in restoring sight. The opaque lens of the eye is removed and replaced by an artificial intraocular lens [17, 18]. Cataract surgery as such is one of the most cost-effective interventions though cost and clinical effectiveness of different surgical techniques and intraocular lenses (IOLs) vary a lot [19]. In clinical decision-making, interventions are being primarily assessed based on efficacy and safety. However, it is also important to monitor the impact that treatments have on utility, i.e. health-related quality of life (HRQoL) using validated instruments [20, 21]. Utility is a measure of health preference anchored around a value of ‘1’ for perfect health and ‘0’ for dead that is used in calculations of quality-adjusted life years (QALY) [22].

This systematic review aims to evaluate health-related quality of life evidences on effects of different cataract surgeries and intraocular lens implantation on visual function and quality of life in age-related cataract patients. This review also attempts to bring together all the cataract-related HRQoL evidences pertaining to different cataract surgical techniques, different intraocular lenses and cataract-related complications.

## Methods

This protocol has been registered a priori in PROSPERO (#CRD42018092377) [23] and follows the Preferred Reporting Items for Systematic Reviews and Meta-Analyses (PRISMA) guidelines as included in Additional file 1.

### Review question

What are the changes in quality of life and visual function in age-related cataract patients after undergoing a particular type of cataract surgery and intraocular lens implantation?

### Search strategy and information sources

Strategies will be designed to identify all relevant studies for HRQoL among age-related cataract patients. We will search the bibliographic electronic databases like the Cochrane Library including the Cochrane Database of Systematic Reviews (CDSR), Cochrane Central Register of Controlled Trials (CENTRAL), EMBASE, SCOPUS, the NHS Economic Evaluation Database (NHS EED), Health Technology Assessment database, MEDLINE, ClinicalTrials.gov, Current Controlled Trials and World Health Organization International Clinical Trials Registry Platform (WHO ICTRP) systematically. For each database, we will use words and expressions from controlled vocabulary (MESH, EMTREE, and others) and free text searching. There will be no language or date restrictions for the literature search. Results will be managed using Covidence online software to facilitate automatic and manual removal of duplicate records, study screening and selection and record keeping. An example search strategy for EMBASE is given as Additional file 2.

### Criteria for considering studies for this review

The studies will be included based on the following criteria:

#### Inclusion criteria

- Population: adult patients with age-related cataracts without any other ocular comorbidity
- Interventions: phacoemulsification, small-incision cataract surgery (SICS), extracapsular cataract extraction (ECCE), intracapsular cataract extraction (ICCE), rigid lens, foldable lens, monofocal lens, multifocal lens
- Comparators: The comparators for the study would be as given below:
  - Surgery: ICCE, ECCE, SICS, phacoemulsification.
  - Lenses: rigid IOL, foldable IOL, monofocal IOL, multifocal IOL
  - Type of cataract: unilateral cataract, bilateral cataract
- Outcomes: generic quality of life (QoL), HRQoL, vision-related quality of life (VRQoL), visual function (VF), cataract surgery-related complications
- Study design: systematic reviews, meta-analysis, randomised controlled trials (RCTs), original observational studies, case-control studies, cohort studies, cost-effectiveness studies

#### Exclusion criteria

- IOLs for non-age-related cataracts like congenital or paediatric cases
- Studies reporting insufficient data for analysis
- Studies evaluating the association between QoL and causes of visual impairment which are unrelated to cataract
- Studies validating the test questionnaire or construct validation studies
- Other eye disorders/diseases along with cataract
- Narrative review articles, study protocols, opinions, abstracts

### Year of publication and language

Eligibility will not be restricted by year nor by language of publication.

### Inclusion screening process

Studies will be selected for inclusion through a two-stage process. The first stage will be to screen the literature search results (titles and, if present, abstracts) identified by the search strategy to identify all citations that potentially meet the inclusion/exclusion criteria detailed above. The second stage will be a ‘preliminary’ data extraction to aid in the study selection process. Full manuscripts of selected citations that appeared potentially relevant will be obtained and preliminary data extraction will be done in order to ensure that all included studies have sufficient data pertaining to health-related generic quality of life and/or vision-related quality of life (Table 1).

**Table 1:** Preliminary data extraction table to be used for inclusion/exclusion of studies

Serial No.	
Study ID	
Title of study	
Published in year	
Study design	
Patient population	
Intervention	
Comparator (If any)	
Outcome measures (e.g. visual acuity, QoL, VF)	
Instruments used	
Included/excluded	
Reason for exclusion	

All studies will be assessed by two reviewers and checked independently by a third reviewer before taking a final decision. At each stage, any disagreements will be resolved by discussion.

### Data extraction process

Two authors will extract data using a standardised data extraction form (Table 2) in Microsoft Excel, and the results will be compared for differences. Discrepancies in the extracted data will be resolved by discussion, with involvement of a third reviewer if necessary.

**Table 2:** Data extraction table to be used for included studies

Category (study is related to surgical techniques, intraocular lenses, surgical complications or any other aspect of cataract)	
Study ID	
Title of the study	
Year of publication	
Country where the work is done	
Aim of the study	
Study settings	
Study design	
Randomisation method (If RCT^α^)	
Preference-based measures (generic/disease specific)	
Which instrument is used	
Sample size	
Age criteria for patient recruitment	
Patient recruitment details	
Follow up details	
Tariff details	
Modelling details	
Statistical tests used	
Cost-effectiveness analysis (e.g, ICER^β^, NHB^γ^)	
Average age of patients	
Visual function scores Pre-surgery Post-Surgery	
Quality of life scores Pre-surgery Post-surgery	
Results	
Conclusion	
Reviewer’s remarks	

^α^Randomised Controlled trials, β- Incremental cost effectiveness ratio, γ- Net Heath Benefit

^β^Incremental cost-effectiveness ratio

^γ^Net heath benefit

### Critical appraisal strategy

The quality of included studies will be assessed by using a modified checklist recommended by Ara et al. [24] and insufficient detail in the primary studies will be noted under limitations. Three main criteria will be considered for quality assessment (Table 3). The fourth criteria from Ara et al. [24], which is ‘In line with reimbursement agency requirements’ will not be considered as the main aim of this study will be to systematically review cataract-related HRQoL evidences, and we do not intend to use the health state utility values (HSUVs) for a particular reimbursement agency here. Two authors will apply the criteria and it will be checked for differences with any disagreements resolved by consensus and involving a third reviewer wherever necessary.

**Table 3:** Quality assessment checklist used for included studies (modified from Ara et al.) [24]

Criteria	Consideration
Criteria 1. Relevance of the study	
Relevant population	How closely do the patient characteristics in the study match to the patient population we have described in our inclusion criteria (PICO)
Relevant health states	Timing of data collection, e.g. pre-surgery, post-surgery, follow-up intervals. The use of any medications that is likely to have an independent effect on HSUVs (either detrimental or beneficial)
Criteria 2. Quality assessment	
Sample size	This was not considered as exclusion criteria, but the precision of the estimate was assessed
Response rates to the measure used	Are response rates reported and if so, are the rates likely to be a threat to the validity of the estimated HSUVs for the health states?
Loss to follow-up	How large is the loss to follow-up and are these likely to threaten the validity of the estimates?
Missing data	What are the levels of missing data and how are they dealt with? Are there details on the causes of the missing data? Again, could this threaten the validity of the estimates?
Criteria 3. Utility values are measured and valued appropriately	
Appropriate use of valuation method	If valuation methods are used (TTO, SG, DCE, VAS) they are used appropriately? Does the valuation method provide preference based values anchored at 1 as equivalent to full health and 0 as equivalent to dead? Are adequate details of the valuation method provided to allow judgement on appropriateness?
Appropriate use of GPMB	Are adequate details of the PBM method provided (e.g. details given on the version used, the social tariff applied) Was the GPBM delivered as intended? (e.g. wording and response options not changed) Is the measure used for the group it was intended (e.g. is an adult GPBM being used for children? Is EQ-5D-Y used with the adult tariff?)
Appropriate health-state description (vignette)	If a health state is valued using a vignette, can the accuracy of the vignette be established? e.g. the process by which it was derived is described

### Method of data synthesis

Studies finally selected for inclusion in the review will be classified into different categories based on the type of cataract surgery and/or intraocular lens it involves and then each category will be classified into the subcategories based on the type of QoL instrument used in the study. Data will be separately analysed for categories and subcategories accordingly.

We will pool the data for each category by performing a meta-analysis if we get enough studies with comparable outcome measures and with similarities in terms of study design, population, instruments used for measuring the health states, value sets used for assigning utility weights and reporting results. A fixed effects meta-analysis will be applied to obtain the pooled effect size with 95% confidence interval (CI) or else a random effects meta-analysis would be performed (heterogeneous, *τ*2 > 0). The heterogeneity level will be investigated by using *I*^2^ index. An *I*^2^ value of more than 75% will be considered as an indication of significant heterogeneity. If there is evidence for substantial heterogeneity or inconsistency, we will not pool the results. In circumstances where pooling of studies will be deemed inappropriate, we will only provide a qualitative discussion of the findings with tables of findings and a narrative description.

## Discussion

Literature search on cataract-related HRQoL evidences results in a huge number of studies when generic terms for HRQoL are used. Cataract-related studies reporting HRQoL are diverse in terms of study design, type of preference-based measure used (disease-specific or generic measures) and type of instruments used (EQ 5D, Short Form 36, Visual Function Questionnaires like VF-11 and VFQ-14, etc.) as there is no set pattern for reporting HRQoL. The diversity of studies is seen as some compare HRQoL between different types of intraocular lenses (e.g. comparison between monofocal and multifocal lenses) where others compare it between different types of cataract surgical techniques (e.g. comparison between phacoemulsification and small manual incision surgery). Still, others measure the HRQoL of cataract patients before and after surgery without mentioning much the details of type of surgical technique and IOLs used. Against this backdrop, the current study will be a broad review to systematically explore, critically appraise and provide insight to the effects of different types of cataract surgeries and intraocular lens implantations on visual function and quality of life in age-related cataract patients. If adequate evidence is available, this review may provide requisite data for decision modelling for economic evaluation studies. The review will also help in identifying gaps in availability of data that will help planning future modelling studies. As the review attempts to bring together all the cataract-related HRQoL evidences pertaining to different cataract surgical techniques, different intraocular lenses and cataract-related complications, it will also identify gaps in evidences and areas where future research is required.

## Additional files

Additional file 1: PRISMA-P 2015 Checklist. (DOCX 30 kb)

Additional file 2: An example search strategy for EMBASE. (DOCX 19 kb)

## Data Availability

Not applicable.

## Abbreviations

CDSR: Cochrane Database of Systematic Reviews
CENTRAL: Cochrane Central Register of Controlled Trials
DCE: Discrete Choice Experiment
DHR: Department of Health Research
ECCE: Extracapsular cataract extraction
GPBM: Generic preference-based measures
HRQoL: Health-related quality of life
HSUV: Health state utility values
HTA: Health Technology Assessment
HTAIn: Health Technology Assessment in India
ICCE: Intracapsular cataract extraction
ICER: Incremental cost-effectiveness ratio
IOL: Intraocular lens
MESH: Medical Subject Headings
NHB: Net health benefit
NHPS: National Health Protection Scheme
NHS EED: National Health Service Economic Evaluation Database
PBM: Preference-based measures
PICO: Population, Intervention, Comparator, Outcome
QALY: Quality-adjusted life years
QoL: Quality of life
RCT: Randomised controlled trial
RSBY: Rashtriya Swasthya Bima Yojana
SG: Standard gamble
SICS: Small-incision cataract surgery
TTO: Time trade-off
UHC: Universal Health Coverage
VAS: Visual analogue scale
VF: Visual function
VRQoL: Vision-related quality of life
WHO ICTRP: World Health Organization International Clinical Trials Registry Platform
