# Supplementary material for: Effects of cataract surgery and intra-ocular lens implantation on visual function and quality of life in age-related cataract patients: a systematic review protocol
Source: Syst Rev. 2019 Aug 13;8:204. doi: 10.1186/s13643-019-1113-6 (PMC6693150; doi:10.1186/s13643-019-1113-6)
Supplement: Supplementary file 2 — An example search strategy for EMBASE. (DOCX 19 kb) [file 13643_2019_1113_MOESM2_ESM.docx]

**#46**

#10 AND #19 AND #26 AND #37 AND #45

[130](http://www.embase.com.elibpgimer.remotexs.in/)

**#45**

#38 OR #39 OR #40 OR #41 OR #42 OR #43 OR #44

[1,093,866](http://www.embase.com.elibpgimer.remotexs.in/)

**#44**

**'meta analysis'**/exp OR **'meta analysis'**

[245,069](http://www.embase.com.elibpgimer.remotexs.in/)

**#43**

**'systematic review'**/exp OR **'systematic review'**

[263,385](http://www.embase.com.elibpgimer.remotexs.in/)

**#42**

**'cost effectiveness studies'**

[1,727](http://www.embase.com.elibpgimer.remotexs.in/)

**#41**

**'cohort studies'**/exp OR **'cohort studies'**

[477,352](http://www.embase.com.elibpgimer.remotexs.in/)

**#40**

**'case control studies'**/exp OR **'case control studies'**

[167,199](http://www.embase.com.elibpgimer.remotexs.in/)

**#39**

**'original observational studies'**

[33](http://www.embase.com.elibpgimer.remotexs.in/)

**#38**

**'randomized controlled trials'**/exp OR **'randomized controlled trials'**

[197,402](http://www.embase.com.elibpgimer.remotexs.in/)

**#37**

#27 OR #28 OR #29 OR #30 OR #31 OR #32 OR #33 OR #34 OR #35 OR #36

[795,155](http://www.embase.com.elibpgimer.remotexs.in/)

**#36**

**'quality of life'**/exp OR **'quality of life'**

[534,584](http://www.embase.com.elibpgimer.remotexs.in/)

**#35**

**'ind-vfq-33'**

[3](http://www.embase.com.elibpgimer.remotexs.in/)

**#34**

**'qaly'**/exp OR **qaly**

[27,236](http://www.embase.com.elibpgimer.remotexs.in/)

**#33**

**'quality adjusted life year'**/exp OR **'quality adjusted life year'**

[24,746](http://www.embase.com.elibpgimer.remotexs.in/)

**#32**

**'indian vision function questionnaire'**

[11](http://www.embase.com.elibpgimer.remotexs.in/)

**#31**

**'cataract surgery related complications'**

[6](http://www.embase.com.elibpgimer.remotexs.in/)

**#30**

**'visual function'**/exp OR **'visual function'**

[263,266](http://www.embase.com.elibpgimer.remotexs.in/)

**#29**

**'health related quality of life'**/exp OR **'health related quality of life'**

[446,131](http://www.embase.com.elibpgimer.remotexs.in/)

**#28**

**'vision related quality of life'**/exp OR **'vision related quality of life'**

[691](http://www.embase.com.elibpgimer.remotexs.in/)

**#27**

**'generic quality of life'**

[577](http://www.embase.com.elibpgimer.remotexs.in/)

**#26**

#20 OR #21 OR #22 OR #23 OR #24 OR #25

[23,316](http://www.embase.com.elibpgimer.remotexs.in/)

**#25**

**'multifocal lens'**/exp OR **'multifocal lens'**

[852](http://www.embase.com.elibpgimer.remotexs.in/)

**#24**

**'monofocal lens'**

[72](http://www.embase.com.elibpgimer.remotexs.in/)

**#23**

**'foldable lens'**

[98](http://www.embase.com.elibpgimer.remotexs.in/)

**#22**

**'rigid lens'**

[117](http://www.embase.com.elibpgimer.remotexs.in/)

**#21**

**'lens implant'**/exp OR **'lens implant'**

[22,856](http://www.embase.com.elibpgimer.remotexs.in/)

**#20**

**'intra-ocular lens implantation'**

[66](http://www.embase.com.elibpgimer.remotexs.in/)

**#19**

#11 OR #12 OR #13 OR #14 OR #15 OR #16 OR #17 OR #18

[51,376](http://www.embase.com.elibpgimer.remotexs.in/)

**#18**

**icce**

[235](http://www.embase.com.elibpgimer.remotexs.in/)

**#17**

**'intracapsular cataract extraction'**/exp OR **'intracapsular cataract extraction'**

[834](http://www.embase.com.elibpgimer.remotexs.in/)

**#16**

**ecce**

[896](http://www.embase.com.elibpgimer.remotexs.in/)

**#15**

**'extracapsular cataract extraction'**/exp OR **'extracapsular cataract extraction'**

[3,376](http://www.embase.com.elibpgimer.remotexs.in/)

**#14**

**sics**

[772](http://www.embase.com.elibpgimer.remotexs.in/)

**#13**

**'small incision cataract surgery'**/exp OR **'small incision cataract surgery'**

[643](http://www.embase.com.elibpgimer.remotexs.in/)

**#12**

**'phacoemulsification'**/exp OR **'phacoemulsification'**

[14,991](http://www.embase.com.elibpgimer.remotexs.in/)

**#11**

**'cataract surgery'**/exp OR **'cataract surgery'**

[49,871](http://www.embase.com.elibpgimer.remotexs.in/)

**#10**

#3 AND #9

[40,477](http://www.embase.com.elibpgimer.remotexs.in/)

**#9**

#4 OR #5 OR #6 OR #7 OR #8

[8,598,659](http://www.embase.com.elibpgimer.remotexs.in/)

**#8**

**'very elderly'**/exp OR **'very elderly'**

[166,462](http://www.embase.com.elibpgimer.remotexs.in/)

**#7**

**'aged'**/exp OR **'aged'**

[4,223,620](http://www.embase.com.elibpgimer.remotexs.in/)

**#6**

**'middle aged'**/exp OR **'middle aged'**

[1,577,571](http://www.embase.com.elibpgimer.remotexs.in/)

**#5**

**'young adult'**/exp OR **'young adult'**

[317,992](http://www.embase.com.elibpgimer.remotexs.in/)

**#4**

**'adult'**/exp OR **'adult'**

[8,318,847](http://www.embase.com.elibpgimer.remotexs.in/)

**#3**

#1 OR #2

[100,160](http://www.embase.com.elibpgimer.remotexs.in/)

**#2**

**'cataract'**/exp OR **'cataract'**

[100,147](http://www.embase.com.elibpgimer.remotexs.in/)

**#1**

**'age-related cataracts'**

[298](http://www.embase.com.elibpgimer.remotexs.in/)
